# Supplementary figures and images for: Striking between-population floral divergences in a habitat specialized plant
Source: PLoS One. 2021 Jun 28;16(6):e0253038. doi: 10.1371/journal.pone.0253038 (PMC8238184; doi:10.1371/journal.pone.0253038)

A

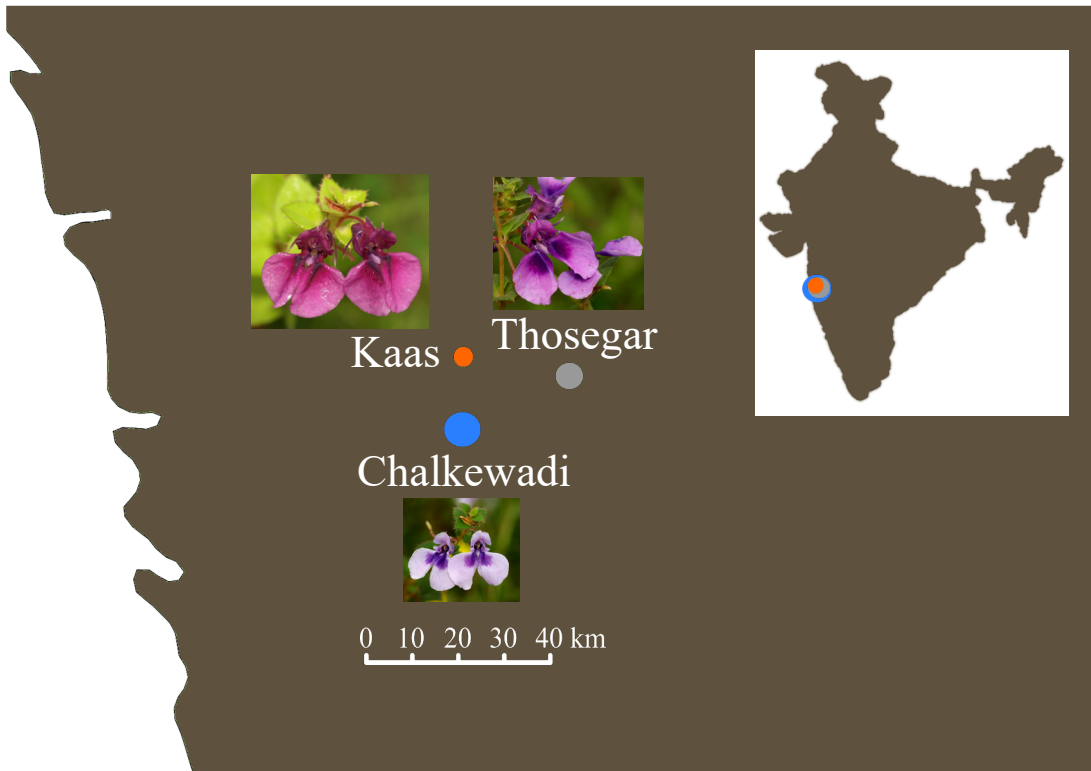

B

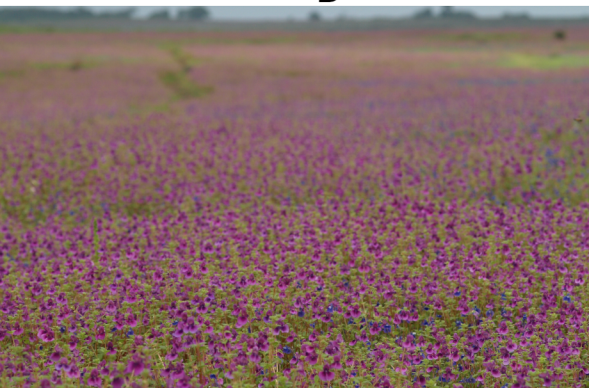

C

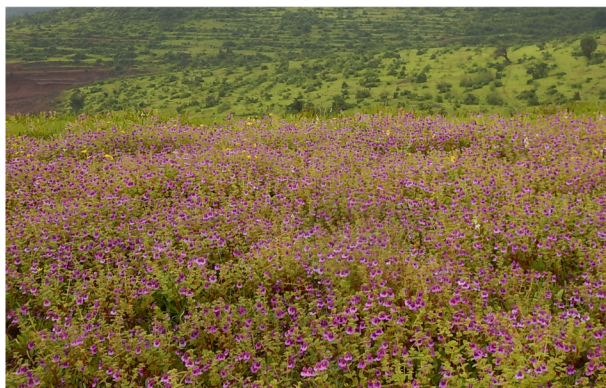

D

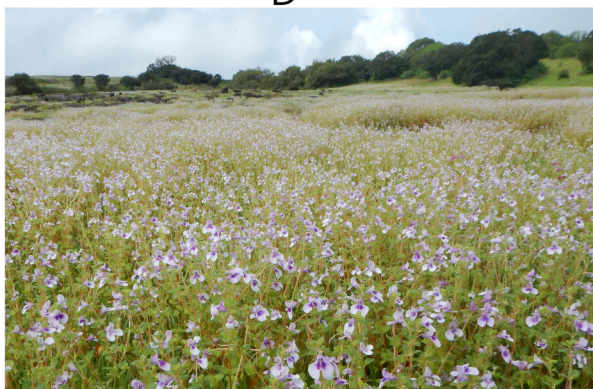

Supplement: S1 Fig — (A) Map showing the location of the study plateaus within the study area, with the inset indicating the location of the study area within India. Flower images are representative of flower morphology across the three populations. Field images of I. lawii from: (B) Kaas; (C) Thoseghar and, (D) Chalkewadi plateaus during the peak flowering season. (PDF) [file pone.0253038.s001.pdf]

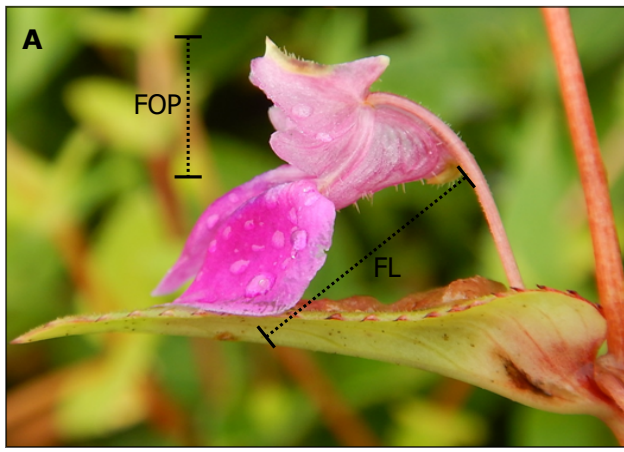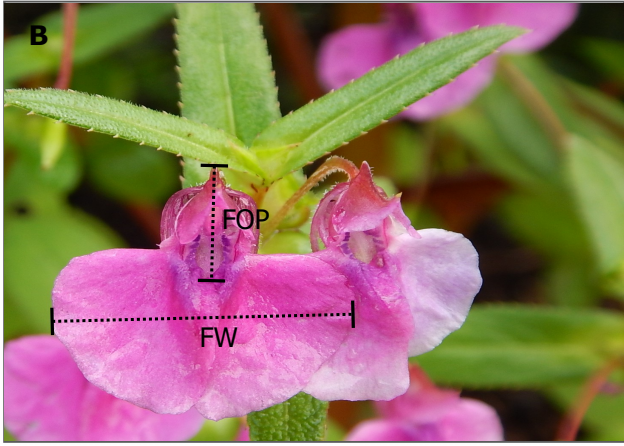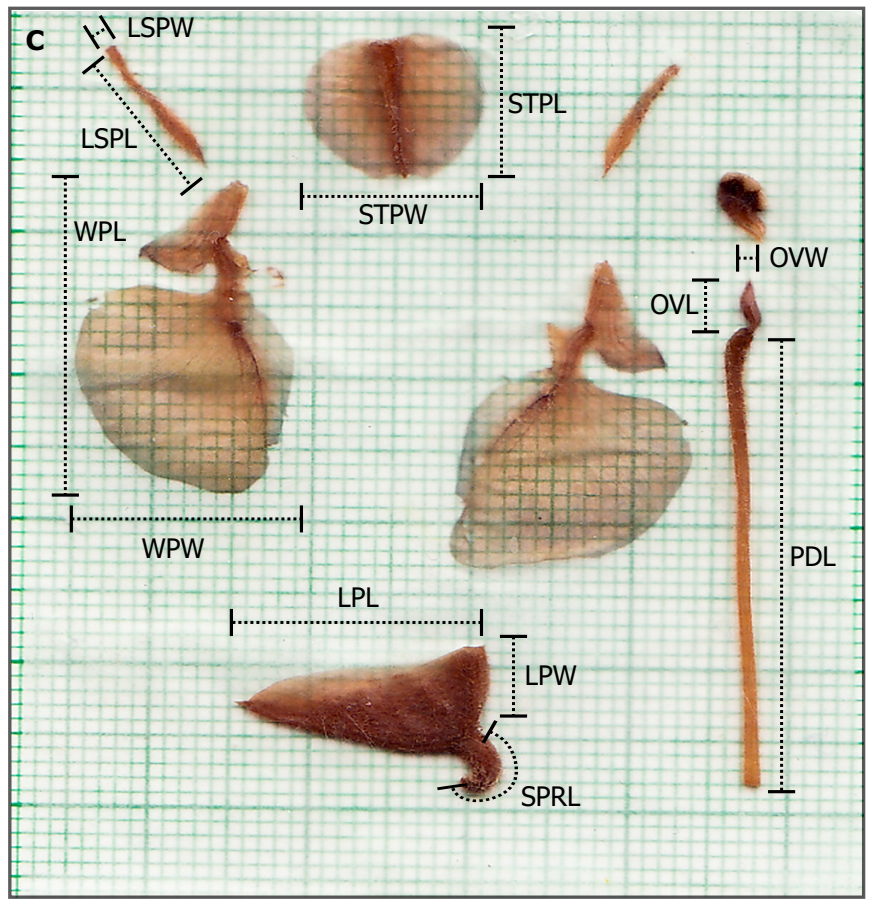

Supplement: S2 Fig — (A) Side view of the whole flower; and, (B) Front view of the flower, depicting the three-dimensional characters measured. (C) Quantification of dissected floral parts. Refer to Table 1 for the details of character abbreviations. (PDF) [file pone.0253038.s002.pdf]

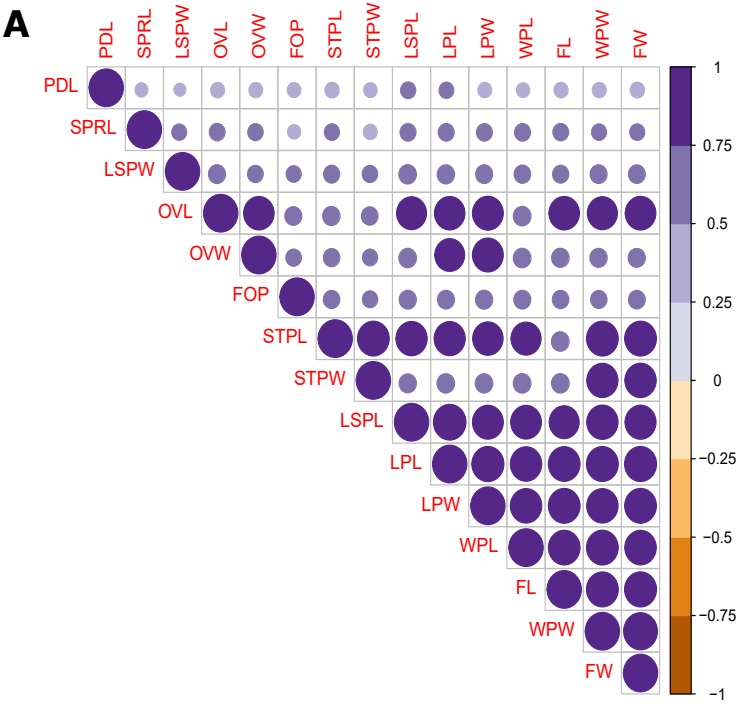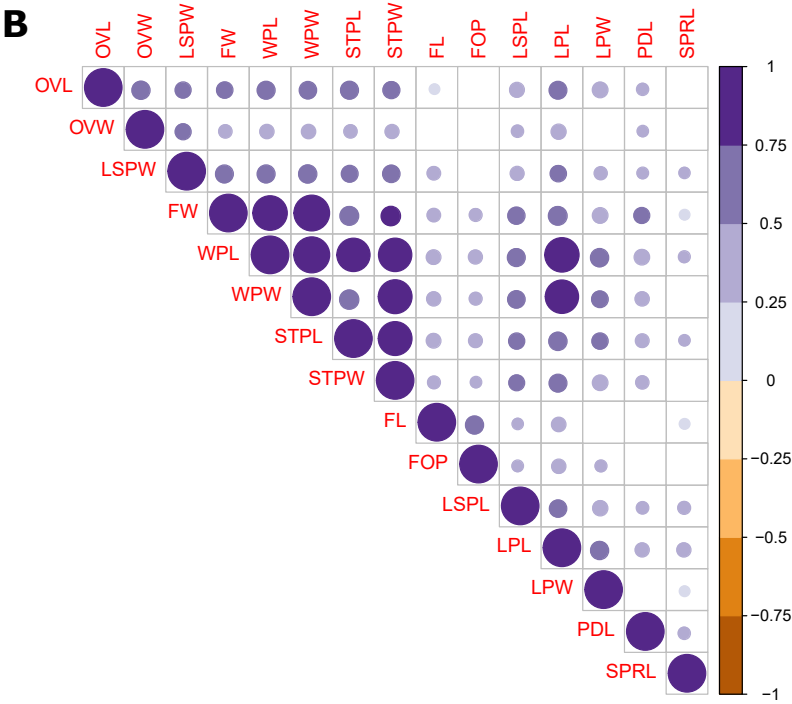

Supplement: S3 Fig — Graph of a correlation matrix made using corrplot() R function highlighting the most correlated variables in a data table in (A) I. lawii; and, (B) I. oppositifolia. Positive correlations displayed in shades of blue and negative correlations in red. The colour intensity and the size of the circle are proportional to the value of correlation coefficients which range from -1 to 1. The legend colour on the right side of the correlogram shows the correlation coefficients and the corresponding colours. See Table 1 for variable names. (PDF) [file pone.0253038.s003.pdf]
