# Supplementary material for: Striking between-population floral divergences in a habitat specialized plant
Source: PLoS One. 2021 Jun 28;16(6):e0253038. doi: 10.1371/journal.pone.0253038 (PMC8238184; doi:10.1371/journal.pone.0253038)
Supplement: S1 Table — Summary of generalized linear models (GLMs) for flower size (FW) for the wild and common garden experiments for a) I. lawii; and, b) I. oppositifolia. The GLM coefficient is reported with the estimate, standard error and p-value generated from the single-step method. Significance is indicated by *** for p < 0.001 and + for 0.05. (DOCX) [file pone.0253038.s004.docx]

**S1 Table**: Summary of generalized linear models (GLMs) for flower size (FW) for the wild and common garden experiments for: a) *I.lawii*; and, b) *I.oppositifolia.* The GLM coefficient is reported with the estimate, standard error and *p*-value generated from the single-step method. Significance is indicated by *** for p < 0.001 and + for 0.05.

|  | |  | **Estimate** | **Std. Error** | **t value** | **Pr(>\|t\|)** |  |
| --- | --- | --- | --- | --- | --- | --- | --- |
| **a) *I. lawii*** | | | | | | | |
| Wild population | | | | | | | |
|  | (Intercept) | | 14.72 | 0.42 | 34.73 | <2e-16 | *** |
|  | Kaas | | 6.98 | 0.60 | 11.55 | <2e-16 | *** |
|  | Thoseghar | | 7.08 | 0.60 | 11.71 | <2e-16 | *** |
| Common Garden (from seeds) | | | | | | | |
|  | (Intercept) | | 10.17 | 0.49 | 20.85 | <2e-16 | *** |
|  | Kaas | | 8.06 | 0.69 | 11.69 | <2e-16 | *** |
|  | Thoseghar | | 7.01 | 0.69 | 10.16 | <2e-16 | *** |
| Common Garden (Transplant) | | | | | | | |
|  | (Intercept) | | 12.80 | 0.51 | 24.90 | < 2e-16 | *** |
|  | Kaas | | 7.88 | 0.73 | 10.84 | < 2e-16 | *** |
|  | Thoseghar | | 6.76 | 0.73 | 9.29 | 0.00 | *** |
|  |  | |  |  |  |  |  |
| **b) *I. oppositifolia*** | | | | | | | |
| Wild population | | | | | | | |
|  | (Intercept) | | 20.02 | 0.60 | 33.19 | <2e-16 | *** |
|  | Kaas | | -1.43 | 0.85 | -1.68 | 0.10 |  |
|  | Thoseghar | | -0.44 | 0.85 | -0.52 | 0.61 |  |
| Common Garden (from seeds) | | | | | | | |
|  | (Intercept) | | 19.44 | 0.46 | 41.92 | <2e-16 | *** |
|  | Kaas | | -0.09 | 0.65 | -0.14 | 0.88 |  |
|  | Thoseghar | | -0.36 | 0.66 | -0.56 | 0.57 |  |
| Common Garden (Transplant) | | | | | | | |
|  | (Intercept) | | 18.16 | 0.40 | 45.16 | <2e-16 | *** |
|  | Kaas | | -0.04 | 0.57 | -0.07 | 0.94 |  |
|  | Thoseghar | | 1.12 | 0.56 | 1.98 | 0.05 | + |
