# Supplementary material for: Striking between-population floral divergences in a habitat specialized plant
Source: PLoS One. 2021 Jun 28;16(6):e0253038. doi: 10.1371/journal.pone.0253038 (PMC8238184; doi:10.1371/journal.pone.0253038)
Supplement: S2 Table — Multiple comparisons of means using Tukey contrasts for flower size (FW) in wild and common garden experiments with: a) I. lawii; and, b) I. oppositifolia. Tukey post hoc multiple contrasts calculated using multcomp package (Hothorn, Bretz, and Westfall 2008) are reported below with estimate, standard error, z value and p-value generated from the single-step method for each pairwise comparison. Significance is indicated by *** for p < 0.001 (DOCX) [file pone.0253038.s005.docx]

**S2 Table.** Multiple comparisons of means using Tukey contrasts for flower size (FW) in wild and common garden experiments with: a) *I.lawii*; and, b) *I.oppositifolia*. Tukey post hoc multiple contrasts calculated using multcomp package (Hothorn, Bretz, and Westfall 2008) are reported below with estimate, standard error, z value and *p*-value generated from the single-step method for each pairwise comparison. Significance is indicated by *** for p < 0.001.

|  | |  | **Estimate** | **Std. Error** | **t value** | **Pr(>\|t\|)** |  |
| --- | --- | --- | --- | --- | --- | --- | --- |
| **a) *I. lawii*** | | | | | | | |
| Wild population | | | | | | | |
|  | Kaas - Chalkewadi | | 6.98 | 0.60 | 11.55 | <1e-06 | ******* |
|  | Thoseghar - Chalkewadi | | 7.08 | 0.60 | 11.71 | <1e-06 | ******* |
|  | Thoseghar - Kaas | | 0.10 | 0.61 | 0.16 | 0.99 |  |
| Common Garden (from seeds) | | | | | | | |
|  | Kaas - Chalkewadi | | 8.06 | 0.69 | 11.69 | <1e-04 | ******* |
|  | Thoseghar - Chalkewadi | | 7.01 | 0.69 | 10.16 | <1e-04 | ******* |
|  | Thoseghar - Kaas | | -1.05 | 0.69 | -1.53 | 0.28 |  |
| Common Garden (Transplant) | | | | | | | |
|  | Kaas - Chalkewadi | | 7.88 | 0.73 | 10.84 | <1e-04 | ******* |
|  | Thoseghar - Chalkewadi | | 6.76 | 0.73 | 9.29 | <1e-04 | ******* |
|  | Thoseghar - Kaas | | -1.13 | 0.73 | -1.55 | 0.27 |  |
|  |  | |  |  |  |  |  |
| **b) *I. oppositifolia*** | | | | | | | |
| Wild population | | | | | | | |
|  | Kaas - Chalkewadi | | -1.43 | 0.85 | -1.68 | 0.21 |  |
|  | Thoseghar - Chalkewadi | | -0.44 | 0.85 | -0.52 | 0.86 |  |
|  | Thoseghar - Kaas | | 0.99 | 0.85 | 1.16 | 0.48 |  |
| Common Garden (from seeds) | | | | | | | |
|  | Kaas - Chalkewadi | | -0.09 | 0.65 | -0.14 | 0.98 |  |
|  | Thoseghar - Chalkewadi | | -0.36 | 0.65 | -0.56 | 0.84 |  |
|  | Thoseghar - Kaas | | -0.27 | 0.65 | -0.41 | 0.90 |  |
| Common Garden (Transplant) | | | | | | | |
|  | Kaas - Chalkewadi | | -0.04 | 0.57 | -0.07 | 1.00 |  |
|  | Thoseghar - Chalkewadi | | 1.12 | 0.56 | 1.98 | 0.12 |  |
|  | Thoseghar - Kaas | | 1.16 | 0.57 | 2.04 | 0.10 |  |
