# Supplementary material for: Striking between-population floral divergences in a habitat specialized plant
Source: PLoS One. 2021 Jun 28;16(6):e0253038. doi: 10.1371/journal.pone.0253038 (PMC8238184; doi:10.1371/journal.pone.0253038)
Supplement: S3 Table — Eigenvalues, variance percent and cumulative variance percent (CVA) derived from Principal Component Analysis (PCA) of the three populations of I. lawii and I. oppositifolia in wild and common garden experiments are included. See Table 1 for variable names. (DOCX) [file pone.0253038.s006.docx]

**S3 Table.** Correlation between floral variables and first three principal component axes with eigenvalues, variance percent and cumulative variance percent (CVA) derived from Principal Component Analysis (PCA) of the three populations of *I.lawii* and *I.oppositifolia* in wild and common garden experiments. See Table 1 for variable names.

| **Species** | **Trait** | **Wild Populations** | | |  | **Germination Experiment** | | |  | **Transplant Experiment** | | |  |
| --- | --- | --- | --- | --- | --- | --- | --- | --- | --- | --- | --- | --- | --- |
|  |  | PC1 | PC2 | PC3 |  | PC1 | PC2 | PC3 |  | PC1 | PC2 | PC3 |  |
| a) *I. lawii* | WPL | -0.91 | 0.1 | 0.07 |  | 0.95 | -0.17 | 0.04 |  | 0.94 | -0.15 | 0.02 |  |
|  | WPW | -0.93 | 0.2 | -0.1 |  | 0.93 | -0.22 | 0.1 |  | 0.92 | -0.22 | 0.06 |  |
|  | STPL | -0.89 | 0.15 | -0.2 |  | 0.91 | -0.19 | 0.07 |  | 0.91 | -0.23 | -0.1 |  |
|  | STPW | -0.83 | 0.27 | -0.3 |  | 0.83 | -0.34 | 0.10 |  | 0.84 | -0.34 | -0.14 |  |
|  | LSPL | -0.91 | -0.07 | -0 |  | 0.88 | 0.07 | -0.09 |  | 0.88 | 0.08 | 0.05 |  |
|  | LSPW | -0.76 | 0.04 | 0.28 |  | 0.75 | 0.01 | -0.38 |  | 0.75 | 0.13 | 0.2 |  |
|  | OVL | -0.87 | -0.07 | 0.16 |  | 0.80 | 0.43 | -0.03 |  | 0.85 | 0.36 | 0.05 |  |
|  | OVW | -0.82 | -0.17 | 0.23 |  | 0.65 | 0.53 | -0.36 |  | 0.71 | 0.48 | 0.16 |  |
|  | PDL | -0.56 | -0.64 | -0.5 |  | 0.46 | 0.41 | 0.73 |  | 0.51 | 0.25 | -0.81 |  |
|  | LPL | -0.94 | .000 | 0.01 |  | 0.94 | 0.05 | -0.02 |  | 0.94 | 0.05 | 0.05 |  |
|  | LPW | -0.92 | 0.03 | 0.09 |  | 0.91 | 0.13 | -0.01 |  | 0.92 | 0.13 | 0.09 |  |
|  | SPRL | -0.67 | -0.37 | 0.39 |  | 0.66 | 0.36 | 0.01 |  | 0.67 | 0.38 | 0.04 |  |
|  | FL | -0.9 | 0.06 | 0.01 |  | 0.92 | -0.19 | -0.02 |  | 0.92 | -0.13 | 0.07 |  |
|  | FW | -0.94 | 0.17 | -0.1 |  | 0.93 | -0.22 | 0.1 |  | 0.84 | -0.24 | 0.13 |  |
|  | FOP | -0.76 | -0.04 | -0.1 |  | 0.89 | -0.16 | -0.04 |  | 0.91 | -0.22 | -0.13 |  |
|  | EV | 10.76 | 0.77 | 0.71 |  | 10.53 | 1.12 | 0.85 |  | 10.63 | 0.97 | 0.8 |  |
|  | Var % | 71.76 | 5.14 | 4.74 |  | 70.23 | 7.46 | 5.66 |  | 70.84 | 6.49 | 5.34 |  |
|  | CV % | 71.76 | 76.9 | 81.6 |  | 70.23 | 77.69 | 83.35 |  | 70.84 | 77.33 | 82.67 |  |
| b)*I.oppositofolia* | WPL | -0.93 | 0.03 | -0.03 |  | -0.92 | 0.03 | -0.04 |  | -0.93 | -0.02 | 0.01 |  |
|  | WPW | -0.91 | 0.01 | -0.12 |  | -0.9 | -0.1 | -0.02 |  | -0.85 | 0.14 | 0 |  |
|  | STPL | -0.85 | 0.11 | -0.16 |  | -0.9 | 0.11 | 0.06 |  | -0.9 | -0.12 | -0.01 |  |
|  | STPW | -0.84 | -0.01 | -0.12 |  | -0.87 | -0.01 | 0.08 |  | -0.84 | -0.17 | 0.12 |  |
|  | LSPL | -0.62 | 0.11 | 0.34 |  | -0.86 | 0.1 | 0.09 |  | -0.86 | -0.18 | 0.08 |  |
|  | LSPW | -0.76 | -0.26 | -0.02 |  | -0.84 | 0.09 | 0.18 |  | -0.83 | -0.25 | 0.16 |  |
|  | OVL | -0.69 | -0.45 | -0.13 |  | -0.68 | -0.37 | 0.33 |  | -0.65 | 0.28 | 0.21 |  |
|  | OVW | -0.51 | -0.65 | -0.13 |  | -0.53 | -0.64 | 0.35 |  | -0.24 | 0.49 | 0.61 |  |
|  | PDL | -0.5 | -0.17 | 0.5 |  | -0.52 | -0.48 | -0.53 |  | -0.45 | 0.23 | 0.21 |  |
|  | LPL | -0.84 | 0.01 | 0.11 |  | -0.89 | 0 | -0.03 |  | -0.83 | 0.06 | -0.11 |  |
|  | LPW | -0.37 | 0.53 | 0.1 |  | -0.61 | 0.37 | -0.07 |  | -0.35 | 0.65 | -0.36 |  |
|  | SPRL | -0.42 | 0.13 | 0.69 |  | -0.49 | -0.03 | -0.65 |  | -0.37 | 0.3 | -0.6 |  |
|  | FL | -0.61 | 0.39 | -0.22 |  | -0.72 | 0.33 | 0.01 |  | -0.83 | -0.17 | -0.11 |  |
|  | FW | -0.85 | -0.01 | -0.02 |  | -0.81 | -0.12 | -0.17 |  | -0.85 | 0.14 | 0 |  |
|  | FOP | -0.54 | 0.46 | -0.32 |  | -0.72 | 0.41 | 0.1 |  | -0.74 | -0.36 | -0.21 |  |
|  | EV | 7.47 | 1.41 | 1.11 |  | 8.76 | 1.24 | 1.03 |  | 8.13 | 1.22 | 1.07 |  |
|  | Var % | 49.78 | 9.41 | 7.43 |  | 58.39 | 8.26 | 6.87 |  | 54.26 | 8.13 | 7.11 |  |
|  | CV% | 49.78 | 59.19 | 66.63 |  | 58.39 | 66.65 | 73.53 |  | 54.26 | 62.39 | 69.49 |  |
