# Supplementary material for: Striking between-population floral divergences in a habitat specialized plant
Source: PLoS One. 2021 Jun 28;16(6):e0253038. doi: 10.1371/journal.pone.0253038 (PMC8238184; doi:10.1371/journal.pone.0253038)
Supplement: S4 Table — Tukey post hoc multiple contrasts were calculated using the multcomp package (Hothorn et al. 2008), and are reported below with estimate, standard error, z value and p-value generated from the single-step method for each pairwise comparison. Pr(>|z|) reported are for PC1. Asterisks depict significance with ‘***’ for p < 0.001; ‘**’ for p < 0.01; ‘*’ for p < 0.05; and, ‘NS’ for p > 0.05. (DOCX) [file pone.0253038.s007.docx]

**S4 Table.** Results of pairwise comparison of plateaus based on the scores of principal axis components; PC1, PC1+PC2, and PC1+PC2+PC3. Tukey post hoc multiple contrasts were calculated using the multcomp package (Hothorn et al. 2008), and are reported below with estimate, standard error, z value and p-value generated from the single-step method for each pairwise comparison. Pr(>|z|) reported are for PC1. Asterisks depict significance with ‘***’ for p < 0.001; ‘**’ for p < 0.01; ‘*’ for p < 0.05; and, ‘NS’ for p > 0.05.

|  | **Estimate** | **Std. Error** | **z value** | **Pr(>\|z\|)** | **PC1** | **PC1+PC2** | **PC1+PC2+**  **PC3** |
| --- | --- | --- | --- | --- | --- | --- | --- |
| **a) *I.lawii*** |  |  |  |  |  |  |  |
| Wild populations |  |  |  |  |  |  |  |
| Kaas- Chalkewadi | -5.85 | 0.38 | -15.31 | <1e-04 | *** | *** | *** |
| Thoseghar- Chalkewadi | -6.49 | 0.38 | -16.96 | <1e-04 | *** | *** | *** |
| Thoseghar- Kaas | -0.63 | 0.38 | -1.65 | 0.22 | NS | *** | * |
| Germination Experiment |  |  |  |  |  |  |  |
| Kaas- Chalkewadi | 5.86 | 0.42 | 13.91 | <1e-05 | *** | *** | *** |
| Thoseghar- Chalkewadi | 6.02 | 0.42 | 14.30 | <1e-05 | *** | *** | *** |
| Thoseghar- Kaas | 0.16 | 0.42 | 0.38 | 0.92 | NS | *** | *** |
| Transplant experiment |  |  |  |  |  |  |  |
| Kaas- Chalkewadi | 5.91 | 0.41 | 14.54 | <1e-04 | *** | *** | *** |
| Thoseghar- Chalkewadi | 6.17 | 0.41 | 15.19 | <1e-04 | *** | *** | *** |
| Thoseghar- Kaas | 0.26 | 0.41 | 0.65 | 0.79 | NS | *** | * |
| **b) *I.oppositifolia*** |  |  |  |  |  |  |  |
| Wild populations |  |  |  |  |  |  |  |
| Kaas- Chalkewadi | 0.40 | 0.71 | 0.57 | 0.84 | NS | NS | NS |
| Thoseghar- Chalkewadi | -0.53 | 0.71 | -0.75 | 0.73 | NS | NS | NS |
| Thoseghar- Kaas | -0.93 | 0.71 | -1.32 | 0.39 | NS | NS | NS |
| Germination Experiment |  |  |  |  |  |  |  |
| Kaas- Chalkewadi | -0.35 | 0.76 | -0.46 | 0.88 | NS | NS | NS |
| Thoseghar- Chalkewadi | 0.44 | 0.76 | 0.57 | 0.83 | NS | NS | NS |
| Thoseghar- Kaas | 0.80 | 0.76 | 1.04 | 0.55 | NS | NS | NS |
| Transplant experiment |  |  |  |  |  |  |  |
| Kaas- Chalkewadi | -0.14 | 0.77 | -0.18 | 0.98 | NS | NS | NS |
| Thoseghar- Chalkewadi | -0.45 | 0.77 | -0.59 | 0.83 | NS | NS | NS |
| Thoseghar- Kaas | -0.31 | 0.74 | -0.42 | 0.91 | NS | NS | NS |
