# Supplementary material for: Striking between-population floral divergences in a habitat specialized plant
Source: PLoS One. 2021 Jun 28;16(6):e0253038. doi: 10.1371/journal.pone.0253038 (PMC8238184; doi:10.1371/journal.pone.0253038)
Supplement: S5 Table — Flower number was included as a random variable. The regression output table presents coefficients associated with the response variable listed to the left under estimate, standard error associated with these estimates, z value, and the two-tailed p-values that correspond to those z-values and the significance. Asterisks depict significance with ‘***’ for p < 0.001; ‘**’ for p < 0.01; ‘*’ for p < 0.05. (DOCX) [file pone.0253038.s008.docx]

**S5 Table.** Summary statistics for the effect of plateau and species (fixed variables) and their interaction on rate of floral visits by visitors identified at the level of species/ morphotypes in *Impatiens* species. Flower number is included as a random variable. The regression output table presents coefficients associated with the response variable listed to the left under estimate, standard error associated with these estimates, z value, and the two-tailed p-values that correspond to those z-values and the significance. Asterisks depict significance with ‘***’ for p < 0.001; ‘**’ for p < 0.01; ‘*’ for p < 0.05.

| **Species** |  | **Estimate** | **Std. Error** | **z value** | **Pr(>\|z\|)** |  |
| --- | --- | --- | --- | --- | --- | --- |
| *Apis dorsata* | Plateau | 2.221 | 0.601 | 3.698 | 0.000218 | *** |
|  | Species | 2.724 | 0.563 | 4.834 | 0.00000134 | *** |
|  | Flower number | 0.001 | 0.000 | 4.241 | 0.0000223 | *** |
|  | Plateau x Species | -2.297 | 0.643 | -3.570 | 0.000357 | *** |
| *Apis cerana* | Plateau | 0.029 | 0.645 | 0.045 | 0.9642 |  |
|  | Species | 0.930 | 0.491 | 1.893 | 0.0583 |  |
|  | Flower number | 0.000 | 0.000 | -0.034 | 0.9732 |  |
|  | Plateau x Species | -1.435 | 0.715 | -2.007 | 0.0448 | * |
| Amegilla sp. | Plateau | -0.299 | 0.436 | -0.686 | 0.492 |  |
|  | Species | 1.847 | 0.371 | 4.977 | 0.000000646 | *** |
|  | Flower number | 0.000 | 0.000 | 1.469 | 0.142 |  |
|  | Plateau x Species | -1.043 | 0.640 | -1.628 | 0.103 |  |
| Butterfly | Plateau | 0.222 | 0.933 | 0.238 | 0.81205 |  |
|  | Species | -1.410 | 1.055 | -1.337 | 0.18116 |  |
|  | Flower number | -0.002 | 0.001 | -2.044 | 0.04097 | * |
|  | Plateau x Species | 0.463 | 1.331 | 0.348 | 0.72806 |  |
| Brown moth | Plateau | -2.893 | 1.299 | -2.227 | 0.0259 | * |
|  | Species | 1.020 | 0.651 | 1.568 | 0.1169 |  |
|  | Flower number | -0.001 | 0.000 | -1.745 | 0.0809 |  |
|  | Plateau x Species | -0.516 | 1.741 | -0.297 | 0.7668 |  |
| Small white moth | Plateau | -0.299 | 0.436 | -0.686 | 0.492 |  |
|  | Species | 1.847 | 0.371 | 4.977 | 0.000000646 | *** |
|  | Flower number | 0.000 | 0.000 | 1.469 | 0.142 |  |
|  | Plateau x Species | -1.043 | 0.640 | -1.628 | 0.103 |  |
| Hawk moth | Plateau | -1.60179 | 1.889732 | -0.848 | 0.3966 |  |
|  | Species | 2.97105 | 1.64462 | 1.807 | 0.0708 |  |
|  | Flower number | 0.00057 | 0.000562 | 2.429 | 0.0151 | * |
|  | Plateau x Species | -2.17194 | 2.051994 | -1.058 | 0.2898 |  |
| Hover fly | Plateau | 1.508247 | 0.463477 | 3.254 | 0.00114 | ** |
|  | Species | 0.473065 | 0.524693 | 0.902 | 0.36727 |  |
|  | Flower number | 0.000303 | 0.000341 | 0.89 | 0.37353 |  |
|  | Plateau x Species | -0.59756 | 0.610764 | -0.978 | 0.32789 |  |
| Slender fly | Plateau | 0.07618 | 2,51900 | 0 | 0.9999 |  |
|  | Species | 0.69001 | 2,51900 | 0 | 0.9999 |  |
|  | Flower number | 0.36062 | 0.000565 | 2.2 | 0.0278 | * |
|  | Plateau x Species | -0.13593 | 0.82689 | -0.164 | 0.869 |  |
| House fly | Plateau | 1.201984 | 1.012268 | 1.187 | 0.23506 |  |
|  | Species | 0.997445 | 0.940975 | 1.06 | 0.28914 |  |
|  | Flower number | 0.000756 | 0.000287 | 2.633 | 0.00848 | ** |
|  | Plateau x Species | -2.31351 | 1.265137 | -1.829 | 0.06745 |  |
| Small fly | Plateau | 1.379759 | 0.59364 | 2.324 | 0.02011 | * |
|  | Species | -0.00945 | 0.585401 | -0.016 | 0.98713 |  |
|  | Flower number | 0.000659 | 0.000464 | 1.419 | 0.15594 |  |
|  | Plateau x Species | 0.733728 | 0.856893 | 0.856 | 0.39185 |  |
| Beetle | Plateau | -1.24336 | 0.679102 | -1.831 | 0.0671 |  |
|  | Species | -0.67586 | 1.077962 | -0.627 | 0.5307 |  |
|  | Flower number | 0.001206 | 0.000447 | 2.697 | 0.007 | ** |
|  | Plateau x Species | -1.20698 | 1.323647 | -0.912 | 0.3618 |  |
