# Supplementary material for: Striking between-population floral divergences in a habitat specialized plant
Source: PLoS One. 2021 Jun 28;16(6):e0253038. doi: 10.1371/journal.pone.0253038 (PMC8238184; doi:10.1371/journal.pone.0253038)
Supplement: Attachment — Submitted filename: Review.docx [file pone.0253038.s012.docx]

Regarding introduction structure can be used as a reference: Yun and Kim 2021 https://doi.org/10.1371/journal.pone.0249752 or more specifically, something like that:

Specialized species > sky islands > Western Ghats> Impatiens genus > floral morphology variation > causes of variation > study species and aims/questions/hypotheses (See structure suggested below)

Many species are specialized in their ecology - for e.g., they use a small subset of the habitats available to them or interact with only a few species among the many they can potentially interact with. The role of ecological specialization in driving diversification of lineages has been widely appreciated [1–4]. Habitat specialization, wherein a species has evolved adaptations to survive only in a particular habitat (or a few habitats), is one axis of specialization with potentially strong consequences for diversification. When the habitat occupied by a specialized species is patchily distributed, gene flow between the fragmented populations can be restricted, allowing local evolution which can result in divergences between populations, and such divergences can lay the foundation for eventual speciation [5–7].

Species adapted to terrestrial insular habitats - called ‘terrestrial islands’ or ‘inselbergs’ - often exhibit high degrees of specialization and habitat fragmentation. ‘Sky islands’ - rock outcrops and other types of high-altitude habitats in mountain systems that are isolated from one another by intervening valleys with markedly different environments - are such terrestrial island habitats. **Sky islands systems across the world have been shown to be hotspots of diversification [8–20].** Examples **of sky islands systems** include the granitic outcrops of South American Atlantic rainforest [8], the rocky grassland fields (campos rupestres) of Brazil, the Madrean woodlands of southern USA and northern Mexico [10]**,** the Eastern Afromontane Biodiversity Hotspot [11], **and tropical montane cloud forests (sholas) in the Western Ghats of southern India [9].**

**The Western Ghats mountains also consist of a** high altitude laterite rich rocky plateaus - flat topped rock outcrops. These are habitats with extreme environmental conditions such as high temperatures during summer and heavy rains during the monsoon, along with strong wind, high diurnal temperature variation, high evapotranspiration and impermeable soils [28, 29]. The microenvironment in rocky plateau habitats differ from that in the surrounding habitats, making the plateaus terrestrial islands.

Several *Impatiens* species are found **in these plateaus terrestrial islands and it can be considered a hotspot of diversification of the *Impatiens* genus**. *Impatiens* (**Balsaminaceae family**), is one of the most species‐ rich genera of angiosperms, with over 1000 species distributed primarily in the Old World tropics and subtropics [21], with a few species also found in the temperate regions [22]. The genus is the most speciose angiosperm genus in India, with > 220 species, and exhibits exceptional diversity and endemism in the Western Ghats mountains [23] with more than 120 species [24], of which > 90% are endemic to these mountains, and new species continue to be reported at a high rate [25–27].

**The species diversification can be result of** morphological variation between populations of a species**, such as morphological variation of floral traits. The morphological variation** can stem from two causes - genetic differentiation and phenotypic plasticity. When the homogenizing effect of gene flow is reduced, adaptations to the local environment, i.e., diversifying selection, can drive genetic differentiation [33]. **FLORAL TRAITS VARIATION AND POLLINATOR-MEDIATED SELECTION HYPOTHESIS??? MATING SYSTEM AND GENE FLOW???**

**On the other hand,** even in the absence of local adaptation, genetic drift in combination with low gene flow can lead to divergences; the ‘isolation-by-distance’ model [34]. ~~Thus, reduction in gene flow in conjunction with local adaptation and/or genetic drift may underlie genetic variation between plateaus in I. lawii.~~ Alternatively, ~~there may be low genetic variation among the three populations, but~~ differences in environments ~~across plateaus~~ may generate varying phenotypes due to phenotypic plasticity. Many plants display strong variation in floral traits as a result of their sensitivity to edaphic or climatic factors [35], although this plasticity may not be adaptive in all cases.

**Here, we investigate diversification of *Impatiens lawii*** **Hook. f. & Thomson,** a plant specialized on the **Western Ghats** rocky plateaus [30, 31]. Our preliminary field observations suggested striking variation in floral morphology across populations (S1 Fig; see Supporting Information with this article) in the Satara plateau cluster which comprises the Kaas, Thoseghar and Chalkewadi plateaus, all within an altitudinal range of 1145-1230 m above 90 mean sea level**. In addition,** **our extensive field surveys confirmed previous reports that *I. lawii* is absent from the low-lying areas. The region has been shaped by Deccan trap volcanism ca. 65 million years ago [28, 32]. Although we cannot be certain that the species was historically not more widespread, it is likely that gene flow between the populations has been restricted to some degree, at least in the recent past. Thus, reduction in gene flow in conjunction with local adaptation and/or genetic drift may underlie genetic variation between plateaus. Otherwise,** we surmise that the morphological variation observed may be part of the reaction norm to a particular environmental variable (or variables) differing among plateaus. **Here we confirm and characterize this variation using morphometric analyses, and investigate the factors underlying the patterns of variation.** QUESTIONS/HYPOTHESES??? POLLINATORS?

We designed experiments to test the **genetic differentiation and phenotypic plasticity** competing hypotheses. In addition to *I. lawii*, we characterized variation in *I. oppositifolia*, a congeneric species found on the three plateaus as well as the intervening low-lying regions. Being a relative habitat generalist, *I. oppositifolia* served as a ‘control’ in our study. We conducted common garden experiments wherein early-stage transplants, and plants propagated from seeds, were reared in an environmentally controlled greenhouse. If phenotypic plasticity generates the differences in phenotype across plateaus, there should be no morphological differences under these common garden conditions. On the other hand, if genetic divergences underlie the morphological variation seen, the differences should also manifest under the common garden conditions.
