# Supplementary material for: Striking between-population floral divergences in a habitat specialized plant
Source: PLoS One. 2021 Jun 28;16(6):e0253038. doi: 10.1371/journal.pone.0253038 (PMC8238184; doi:10.1371/journal.pone.0253038)
Supplement: Attachment — Submitted filename: Response to Reviewers_R1.docx [file pone.0253038.s013.docx]

We thank both reviewers for their critical evaluation and comments, which have improved the quality of the manuscript. We have incorporated all their suggestions. The referee comments and our point-by-point responses are below. Our responses are preceded by ‘###’

**Reviewer #1:**

The present study presents interesting and robust results about habitat specialization with regard to species diversification. In this respect, the comparative analysis with *I. oppositifolia,* the experiments in common gardens and the analysis of floral visitors, give support substantiated to the conclusions.
That said, the following analysis basically focuses on the structuring of the manuscript.

Introduction
Some fragments of this item are allocated inappropriately. For example: page 5, lines 86 to 91 ("Our preliminary field observations suggested ..." to "... underlying the patterns of variation), it is characterized more as results than" Introduction "itself.

\### We agree that, upon first read, this part may seem more appropriate for Results than for Introduction. We considered deleting this or moving it elsewhere. However, we decided not to, because of several reasons. First, we honestly would not have undertaken this study if not for the striking variation we saw across plateaus. Second, unless readers know that there are floral differences across the plateaus, they will be unable to appreciate the hypotheses/predictions made in the last paragraph of the revised version. Third, Reviewer 2 suggested a major restructuring of Introduction. In this revised structure, it is even more useful/important to mention this.

The third paragraph on page 12, lines 112 to 120 would be more appropriately allocated to the methodological description item.

\## This paragraph (now, the last paragraph of Introduction) has been extensively revised, based on suggestions by the Reviewer 2. We have deleted or moved to the results a major part of this section (L115 to L120) to the Methods section. We retain only the initial part (L112 to L114) in the Introduction “In addition to *I. lawii*, we characterized variation in *I. oppositifolia*, a congeneric species found on the three plateaus as well as the intervening low-lying regions. Being a relative habitat generalist, *I. oppositifolia* served as a ‘control’ in our study”, because this part is needed for readers to understand that we had a generalist control. Importantly, we now have explicit hypotheses involving a comparison between the generalist and specialist species, and, therefore, this information is crucial and cannot be moved to Methods.

It is also suggested, at the end of the Introduction item, to clearly and objectively redefine the objectives of this study. For example: "... considering these factors, we aim to quantified pollinator visitation across plateaus through field surveys."

\### This was an excellent suggestion. The last paragraph of Introduction now has clear hypotheses/questions of the study

Methods
The sub-item “Study species” is information regarding the introduction. The item “Methods” must be described in a concise, objective and encompassing determinants listed by the authors in a way that understands the tools used for the development of this study.

\### We agree with the reviewer. The Introduction already has a brief description of the study species. This section in the Methods was meant to provide more detailed ecological information about the study species, but was out of place. We have moved some of the additional details to the Introduction, and deleted the rest since they are not important in the context of the study. As a consequence, the manuscript is more concise and reads better.

Considering the methodology used to define the topic “Pollinator visitation”, I recommend changing to flower visitors as presented on Results.

\### This is a good suggestion. We have changed the title to ‘Floral visitation’, and this also matches the title in the Results section.

**Reviewer #2**:

General comments

This paper describes the floral traits variation patterns of two species on three plateaus that form sky islands in India. The study highlights the importance of habitat specialization and sky islands in diversification of plants. The paper is innovative in showing that populations of habitat specialized species in sky islands areas, although a very close areas, without any difference on altitude, latitude and climatic factors differ in floral traits and therefore could be led to diversification.
There are three main implicit questions in the manuscript:

“Do floral traits vary similarly for specialized/regional species and non-specialized/widespread distribution species on sky islands?
Which factor best explains the variation on floral traits in these species - phenotypic plasticity or genetic differentiation?
Can floral traits divergence among populations of each species be related to pollinators?”
The aims/hypotheses/questions should be more explicit at the end of introduction section, and the question and hypothesis concerning to floral traits and pollinators need to be defined clearer, as well as the analyses to test it (see comments below).
\### This is an excellent suggestion. The last paragraph of Introduction now has explicit hypotheses/predictions.

The authors directly test the phenotypic plasticity and indirectly the genetic differentiation. Although genetic differentiation has not been directly tested (using usual genetic measures such as fixation index Fst or Nei’s Gst), the paper contains a large amount of carefully collected data and suggestive results of genetic differentiation.
Also, there is no mention of the mating system of plant species and its populations. Maybe information about it could be help to explain some results (see details below and the paper: Gamba and Muchhala 2020 10.1111/mec.15575). It should at least be discussed.

\### Based on the Gamba and Muchhala 2020 reference suggested, and other references, we have now added a brief discussion on the effects of pollination and mating systems on genetic differentiation in plants (L 402 to L 411).

As mentioned by the authors in the discussion section, not all floral visitors are pollinators, can be herbivores, thieves, occasional pollinators, effective pollinators, but the term “floral visitors” is being used interchangeably as a synonym for pollinators in the manuscript (see details below). It needs to be revised.

\### We agree with the reviewer. We have done the following

a) In the last paragraph of Introduction and the subtitle of Methods, we have changed ‘Pollinator visitation’ to ‘Quantification of floral visitors’. We have changed ‘pollinators’ to ‘floral visitors’ in the Abstract.

b) In the Methods and Results sections, we use the term ‘floral visitors’ and never ‘pollinators’. An exception is when we present results from the PCA analyses of floral traits, where we mention that some sets of traits are thought to be associate with pollinator attraction and efficiency (supported by references).

c) We deleted Lines 375-377 which talked about pollinator abundance.

d) In the legend to Fig 5, we changed ‘The number of floral visitors belonging to the different pollinator groups’ to ‘The number of floral visitors belonging to the different putative pollinator groups’.

e) In Discussion, we have already included a section about caveat that we have data on floral visitors but not on pollinators (L 436)

f) We changed “pollinator abundance was similar” to “floral visitation rates were similar” (L 415)

Number of visitors (abundance), number of visits and visitation rate are also merged in the manuscript.

\##	We acknowledge that we had unintentionally used number of visitors (implying abundance), number of visits and visitation rate interchangeably. We quantified the number of visitors per unit time - we clarify this clearly now in Methods. Therefore visitation rate is the most appropriate term, and we now use this consistently wherever applicable (particularly in the results section when describing our actual data). However, we retain number of visitors in some parts in Discussion where this reads better. We believe that visitation rates and number of visitors are can be used interchangeably here because our observation periods across plateaus and species are similar and cover the diurnal activity periods for these floral visitors.

Finally, although populations and species share a similar community of floral visitors (composition), the authors show that the abundance and probably the efficiency change. Despite specific and complex cases herbivores can indirectly influence the floral traits variation, which are mainly affected by abundance and efficiency of pollinators (see Nattero and Cocucci 2007 10.1111/j.1095-8312.2007.00756.x). Furthermore, species seem to show phenotypic integration in floral traits, which is also mediated by pollinators (see Ellis et al 2014 https://doi.org/10.1098/rstb.2013.0563). Thus, pollinator-mediated selection as driving floral traits divergence cannot be considered completely unlikely.

\## We agree with the reviewer, and we have already acknowledged that we cannot comprehensively rule out pollinator mediated selection driving the observed differences in floral traits. We now, have further modified the language to emphasize this (L 432).

In summary, this paper includes a lot of interesting and new data to the topic. However, clarifying some issues and some additional information are needed to corroborate the conclusions.

Introduction:
The introduction has relevant information that is required to understand the general topic. Some restructuring should be done to clarify the study proposal and to provide smooth reading, and more information added to explain the expected relation between pollinators and floral traits variation in this study.

Line 1 – “for e.g.,” is redundant. Choose between “for example” or “e.g.”. Actually, in this case “i.e.,” is better than others.

\### It appears that the reviewer refers to first sentence of the Introduction. We have now changed this to ‘for example’.

Line 65 – The seminal study is missing, which coined the term “sky islands” - Heald, Weldon F. 1951. Sky Islands of Arizona. Natural History, 60: 56-63, 95-96.

\### Thank you for pointing this out. We have cited this reference now (L 60).

Line 88 – Fig S1 – Image from Thoseghar plateau is missing.

\### We have now added an image representing flowers from Thoseghar

Line 122 – Floral traits play important roles in visitation by pollinators, but the floral traits evolution and therefore its differentiation can also be mediated by pollinators.

\### We have added ‘...in turn, pollinators can also influence floral trait evolution’ (L 89)

Regarding structure can be used as a reference: Yun and Kim 2021 https://doi.org/10.1371/journal.pone.0249752 or more specifically, something like that:
Specialized species > sky islands > Western Ghats> Impatiens genus > floral morphology variation > causes of variation > study species and aims/questions/hypotheses (See structure suggested in the attached file)
\### We have revised the Introduction based on the structure suggested by the Reviewer.

Methods:
Study species:
– Population sizes? Both species are herbs? Breeding system? Mating system? It is a key information.
\### Reviewer 1 suggested that the subsection ‘Study species’ was inappropriate in the Methods section and that this should be discussed in the Introduction. The study species have been briefly described in the Introduction, and we have now added some information about the habit (both species are herbaceous) and the breeding/mating system (L 101).

Floral variation in natural populations:
Line 145 – “The study species are not cleistogamous”. It can be moved to “study species section”, along with the description of species reproductive system.

\### Please see our response to the previous point. We now mention that the species are not cleistogamous in the Introduction (L 101)

Line 146 – We collected fully opened flowers during the peak flowering season It is duplicated (lines 144-145 and 149-151). It can be replaced by:
Whole individuals were collected and stored in sealed plastic bags and one fully opened flower (male or female flowers???) (petals unfolded entirely) was preserved in FAA (Formaldehyde Alcohol Acetic Acid, 10%:50%:5% + 35% water).

\### We have incorporated these changes. First paragraph of subsection ‘Floral variation in natural populations’ in Methods.

Line 156 – Fig 1 and Fig S2 are the same figure.

##Figure 1 is for *I.lawii* and Fig S2 is for *I.oppositifolia*. As *I.lawii* was the focal species in this study we present the information for this species in the main section, and the same information - representative image of the flower and dissected parts in the, for *I.oppositofolia* in the supplementary material.

Line 183 – Table 1 – A column in the table with the association between traits and pollinator efficiency, flower size... would be helpful (it is mentioned only at the results section; lines 294-295).
\## This is a good suggestion, and we have now included this information in Table 1.

Transplant experiments:
Line 193 – How many plants were transplanted per species and population?
##??? We transplanted 30 seedlings each per species per plateau at their 4-leaved stage and with a height of 5-8 cm. This is now mentioned in L 166

Pollinator visitation:
Line 207 – Why did you not observe the floral visitors of Thoseghar population?

\## Due to logistical constraints we could not simultaneously (within the short period of time coinciding with the peak flowering period of these species) conduct floral visitor observations in all three plateaus. Given these constraints we chose the two farthest plateaus for conducting the floral visitor surveys.

Line 216 – “All insect species that physically touched the flower were counted as a floral visitor”
As mentioned in the general comments, the shifts in floral traits mainly depend on effectiveness and abundance of pollinators.
In the introduction the authors describe: “In order to understand whether floral trait variation across plateaus in *I. lawii* has a functional consequence with respect to pollination, we also quantified pollinator visitation across plateaus through field surveys.” Again, the aim needs to be clarified and standardizing the used term (floral visitors or pollinators). If the hypothesis is based on pollinators community, do analysis only with pollinators and change the affirmative to: “All insect species that physically touched the reproductive structures of flower were counted as a pollinator”.
\### We agree that we have floral visitor data but not pollinator data. We have, therefore, been careful in our interpretation of the floral visitation data. We now state in the Introduction that we quantified floral visitation. In our second response to this reviewers comments where we detail what changes we have made to clearly indicate that we have only measured floral visitation and not pollinator visitation.

Statistical analyses

Line 224 – What are the vegetative traits?
\### This was an error, there were no vegetative traits in this study. We have corrected the sentence (L 199)


Results:
Fig 2 – The letters are missing.
\### Yes, letters representing significant differences were missing. We now represent significant differences using asterices.

Correlation between floral traits and variation in flower size:
Line 277 – The word “letter” is duplicated.
\### We removed the letters representing significant differences .We now represent significant differences using asterices (L248).

Floral variation in natural populations:
Line 292 – Why did you do a second PCA with all floral traits? Since the first PCA is being used to select the best trait for comparison, FW in this case. (Lines 257-258: “FW was strongly correlated with all other floral characters, and we use FW to represent flower size variation across plateaus.”)
\### We did the first analysis examining bivariate relationships to understand the relationships between the floral traits examined. We found that flower width, which is representative of flower size was consistently correlated with most other floral traits examined in both species, and we use this trait to examine variation in size between the study populations. The second analysis (PCA) examined the multivariate relationship between the floral traits. The PCA analysis revealed that in multivariate trait space most of the variation in floral morphology was explained by the first three PC axes. We used these composite variables (PCA1, 2 &3) representing combination of important floral traits to examine variation of overall floral morphology in the study populations.

Floral visitation:
Lines 375-377 – This sentence should be moved to discussion section.
\### Yes, this was inappropriate in the Results section, and we have deleted this. The same has already been discussed in the Discussion section (L 413 to L 425).


Discussion and conclusion:
The authors discussed the results, mentioned the limitations of their study and what could be done in the future to clarify the findings. However, not all statements and conclusions were consistent with introduction and results sections or some relevant information is missing. See suggestions in the comments above.

\### We have responded to all the comments and incorporated the useful suggestions of the reviewer.

Line 411 – “floral morphology differs significantly in all pairwise population comparisons (Table S7, Fig 4A-C)”, except for Thoseghar-Kaas.

\### All three pairwise comparisons (including Thoseghar-Kaas) were indeed significantly different (presented in the first paragraph under ‘Multivariate analyses’ in Results. There was an error in the figure, which we have now corrected
